# Effectiveness of local exercise therapy versus spinal manual therapy in patients with patellofemoral pain syndrome: medium term follow-up results of a randomized controlled trial

**DOI:** 10.1186/s12891-021-04310-9

**Published:** 2021-05-15

**Authors:** Aldo Scafoglieri, Jona Van den Broeck, Stijn Willems, Rob Tamminga, Henk van der Hoeven, Yde Engelsma, Stijn Haverkamp

**Affiliations:** 1 Department of Physiotherapy, Human Physiology and Anatomy (KIMA), Experimental Anatomy Research Group, Vrije Universiteit Brussel, Laarbeeklaan 103, 1090 Brussel, Belgium; 2 SOMT University of Physiotherapy, Softwareweg 5, Amersfoort, BN 3821 The Netherlands; 3 Department of Neuroscience, VU University Medical Center, Amsterdam, HV 1081 The Netherlands; 4 Fysioholland, Medicort, Rijksweg 69, Naarden, GE 1411 The Netherlands; 5 Bergman Clinics BV, Rijksweg 69, Naarden, GE 1411 The Netherlands

**Keywords:** Effectiveness, Exercise, Manipulation, Patellofemoral pain syndrome, Medium term, Randomized controlled trial

## Abstract

**Background:**

Increasing evidence has shown benefits of spinal manipulations in patients with patellofemoral pain syndrome (PFPS). There is scarcity regarding medium term effects of spinal manual therapy on outcome measures in PFPS patients. Therefore, the aim of the present study was to compare the effectiveness of local exercise therapy and spinal manual therapy for knee pain, function and maximum voluntary peak force (MVPF) velocity of the quadriceps in PFPS patients.

**Methods:**

Forty-three patients with PFPS were randomly assigned to a local exercise or spinal manual therapy group. The local exercise group received six sessions (one session per week) of supervised training of the knee-and hip muscles with mobilization of the patellofemoral joint. The spinal manual therapy group received six interventions (one intervention per week) of high velocity low thrust manipulations at the thoracolumbar region, sacroiliac joint, and/or hip. All patients were also asked to do home exercises. Maximum, minimum and current pain were measured using the visual analogue scale. Function was assessed with the anterior knee pain scale (AKPS) and MPFV was recorded using a Biodex System 3 dynamometer. Patients were assessed before intervention, after 6 weeks of intervention and after 6 weeks of follow-up. Between-group differences at assessments were analysed by way of analysis of covariance with Bonferroni correction.

**Results:**

Pain and functionality improved more following spinal manipulative therapy than local exercise therapy. After 6 weeks of intervention the between-group difference (local versus spinal) for maximal pain was 23.4 mm [95% CI: 9.3, 37.6; effect size (ES): 1.04] and − 12.4 [95% CI: − 20.2, − 4.7; ES: 1.00] for the AKPS. At 6 weeks of follow-up the between-group difference for maximal pain was 18.7 mm [95% CI: 1.4, 36.0; ES: 0.68] and − 11.5 [95% CI: − 19.9, − 3.3; ES: − 0.87] for the AKPS.

**Conclusions:**

This study suggests that spinal manual therapy is more effective than local exercise therapy in improving pain and function in patients with PFPS in the medium term. We suggest for future research to investigate whether combining local exercise therapy and spinal manual therapy is more effective than either single intervention on its own.

This clinical trial study was approved by the Medical Ethics Committee METC Z under registration number NL57207.096. and registered retrospectively in ClinicalTrials.gov PRS with registration ID number NCT04748692 on the 10th of February 2021.

**Supplementary Information:**

The online version contains supplementary material available at 10.1186/s12891-021-04310-9.

## Background

Patellofemoral pain syndrome (PFPS) is a chronic condition of the musculoskeletal system characterized by retropatellar and/or peripatellar pain usually worsening during weight-bearing activities [1]. In the general population annual prevalence rates for patellofemoral pain of approximately 25% have been reported [2]. Most patients with PFPS report a feeling of stiffness, especially with knee flexion [3]. Functional activities such as walking, running, jumping, stair climbing and prolonged sitting and kneeling usually increase symptoms [4].

Although not entirely understood, the aetiology of PFPS has been considered multifactorial [5]. Laxity of the knee joint, decreased knee extensor strength, malalignment of the lower extremity and poor coordination between vastus lateralis and vastus medialis obliquus muscle activation have been identified as local risk factors [6–8]. Proximal risk factors such as dysfunction of the lumbosacral region and sacroiliac joint (SIJ), and decreased hip range of motion have also been associated to PFPS [9–12].

The use of exercise therapy has recently been reconfirmed as the intervention of choice in the management of patellofemoral pain [1]. Combined knee and hip targeted exercises have been shown effective in reducing pain and improving function in the short, medium and long term. However, PFPS has also been shown recalcitrant to knee and hip strengthening exercises and may persist for many years [13]. About half of the patients with PFPS continue to experience pain and dysfunction at mid and long term [14, 15]. Due to its persistent nature the absence of full recovery may result in psychological disorders such as a higher mental distress, kinesiophobia, anxiety, catastrophizing and depression [16, 17]. Since patellofemoral pain usually precedes knee osteoarthritis [4] failure of an effective conservative management strategy for PFPS may potentially lead to invasive medical procedures later in life.

Recently, combined exercise and spinal manipulative therapy interventions have been recommended in the treatment of patellofemoral pain in the short and medium terms [1, 18]. Spinal manual therapy may include hands-on mobilisations and/or manipulations of the thoracolumbar region and/or SIJ. Although their immediate positive effects have been repeatedly demonstrated [9, 11, 19–24], lumbar manipulations have been considered inappropriate as stand-alone intervention in patients with PFPS in the short term [1]. To the knowledge of the authors, no earlier studies compared the effectiveness of local exercise therapy with spinal manual therapy in the medium term.

The effectiveness of spinal manual therapy in the treatment of PFPS is based on the concept of regional interdependence of musculoskeletal problems [25]. Since the thoracolumbar zygapophyseal joints (T12 to L3) and knee joint (L2 to S2) share a partial common innervation, it has been suggested that thoracolumbar manipulations appear to modulate afferent input by stimulating inhibitory systems at various spinal levels [26]. Likewise, since the SIJ (L1 to S2) and quadriceps muscle (L2 to L4) share overlap in segmental innervation [27], it has been suggested that SIJ manipulation may increase activation and strength of the quadriceps in patients with PFPS [11, 19]. Hereby, altered mechanoreceptor afferent activity in the ventral part of the SIJ may contribute to a decrease in quadriceps muscle inhibition [19].

Because the effectiveness of spinal manual therapy in the medium term has not been investigated yet, the aim of this study was to compare local exercise therapy with spinal manual therapy in patients with PFPS after 6 weeks of intervention and after 6 weeks of follow-up.

## Methods

### Participants

This study was approved by the Medical Ethics Committee METC Z under registration number NL57207.096. and registered retrospectively in ClinicalTrials.gov PRS with registration ID number NCT04748692 on 10/02/2021. All patients signed a written informed consent form prior to enrollment in our study. Patients diagnosed with PFPS were referred by the orthopaedic surgeons of the Bergman Clinic (Bergman Clinics, Naarden, the Netherlands) to the outpatient physical therapy clinic of Medicort during the period from 2016 through 2019. Adolescents with a minimum age of 16 years and adults were eligible to participate in the study. Minimum age was based on the Tanner scale for physical measurement of development. All patients had non-traumatic anterior knee pain lasting for more than 3 months. The clinical diagnostic criteria for PFPS were: self-reported unilateral or bilateral anterior knee pain provoked by at least two of the following activities: jumping, squatting, ascending/ descending stairs, kneeling, prolonged sitting and or a positive patellar compression test [28]. Exclusion criteria were: experiencing pain for less than 3 months, a history of knee surgery, meniscal lesion, patellar subluxation/dislocation, evidence of tendinopathy or ligamentous pathologies, dislocation or fracture in the pelvic region, spinal surgery, osteoporosis, pregnancy, neurologic disorders or findings of chondromalacia > grade 2 on MRI, echography or X-ray. The inclusion of patients was done by the orthopedic surgeons based on a clinical examination. Medical imaging for diagnostic purposes was also requested by the orthopedic surgeons, usually including X-ray radiography. All patients meeting the inclusion criteria were enrolled by the orthopedic surgeons of the Bergman Clinic.

### Study design and sample size calculation

This study is a randomized controlled trial with a follow-up of 6 weeks. Patients with PFPS were randomly assigned to one of two intervention groups (local exercise group or spinal manual therapy group) using an online computer-based pseudo-random number generator on which the numbers were generated by use of a complex algorithm (seeded by the computer’s clock) [29]. The allocation of the patients occurred before baseline assessment by the outpatient clinic manager.

Sample size calculation was based on the preliminary results of a pilot study in our clinic (*n* = 45). The local exercise therapy group showed a 30% reduction of pain on the visual analogue scale (VAS). A reduction of 50% on the VAS after 6 interventions for the spinal manual therapy group was realized. For a power of 90% and with an alpha of 0.05, the sample size was estimated to be at least 38 patients (19 per group). According to Murphy (1990) [30] a dropout rate between 20 and 40% is to be expected in longitudinal studies of musculoskeletal disorders. By the fact that we requested patients to also be available after their treatment we randomized 62 patients.

### Interventions

The intervention period lasted 6 weeks for both treatment groups. During this 6-week period the local exercise therapy group focused on strengthening knee and hip muscles three times a week following the recommendations of Kooiker et al. (2014). Once a week, patients trained with the support of a physiotherapist. The physiotherapist gradually increased the intensity of the exercises improving muscle endurance. The exercises were supplemented with mobilisations of the patellofemoral joint. Twice a week, patients trained at home following a prescribed exercise program writing down their work-out in an exercise journal.

The spinal manual therapy group was treated once a week for a period of 6 weeks. Before each treatment a qualified manual therapist with 8 years of clinical experience performed a clinical examination of the lower back, SIJ, hip and knee according to the guidelines of van der El (2009) [31]. During the clinical examination the therapist searched for left-right differences in range of motion. Manipulation of a joint was conducted if a movement impairment was found in any of the regions. The movement impairments of the spine were assessed with three dimensional extension of the thoracolumbar spine and joint play of the SIJ. As such, manipulations were only provided in case of a clinical indication. No visits yielded manipulations to different regions other than those described in our manuscript (thoracolumbar region, SIJ or hip joint). Anatomical maps showing innervation areas of spinal nerve roots were used as patient education to explain the regional interdependence model in the treatment of anterior knee pain [10, 25, 32]. Patients were also asked to do home exercises focusing on mobilizing the thoracolumbar region and to write down their performance in an exercise journal (see Supplementary file).

### Outcome measures

The following baseline characteristics were self-reported: age (in years), weight (in kg), height (in cm), duration of symptoms (in months), weekly participation in sport (yes/no), previously receiving exercise therapy treatment for the knee (yes/no). Patellofemoral chondral lesions were graded using the Kellgren and Lawrence system for classification of osteoarthritis (grade 0 = definite absence of X-ray changes of osteoarthritis, grade 1 = doubtful joint space narrowing and possible osteophytic lipping, grade 2 = definite osteophytes and possible joint space narrowing).

Knee pain, functionality and force were measured using validated measurement instruments. Pain and functionality were our primary outcomes. Maximum, minimum and current pain intensity was indicated on a 0–100 mm VAS line. The VAS scale has high test-retest reliability (ICC = 0.97), correlates well with the Kellgren and Lawrence system for classification of osteoarthritis (r = 0.84) and good sensitivity for the measurement of chronic knee pain [33, 34]. Maximum and minimum pain for the previous week was recorded. Functionality was measured using the Dutch version of the anterior knee pain scale (AKPS). The AKPS questionnaire consists of 13 items assessing subjective symptoms and functional limitations totalling a maximum score of 100. Our secondary outcome, maximum voluntary peak force (MVPF) of the quadriceps, was measured using a Biodex system 3 isokinetic dynamometer (Biodex Medical Systems, Inc., Shirley, NY, USA), following the same procedures as described by Hillermann et al. (2006). This system has shown sufficient reliability and validity for position, torque and velocity measurements in clinical and research settings [35, 36]. Patients were assessed before intervention (=baseline), at 6 weeks (=immediately after the last intervention) and at 12 weeks (=6 weeks after the last intervention). The researcher collecting the outcome measures was unaware of the group status of the patients.

### Statistical analysis

IBM SPSS Statistics 25 was used to analyse our data. Shapiro-Wilk tests were used to determine normality of the outcome measures. Baseline results of the local exercise therapy and spinal manual therapy groups were compared using chi-squared tests (for categorical data). independent t-tests (for parametric continuous data) or Mann-Whitney U tests (for nonparametric continuous data). Paired samples t-tests or Wilcoxon signed rank tests were used to check for differences between pre- and post-intervention results within each group. Between-group differences were assessed by way of analysis of covariance with Bonferroni correction. Hereby, differences in pre to post-intervention and follow-up change were calculated with the baseline descriptives put as a covariate in the regression model. The level of significance was set at *p* < 0.05 and adjusted mean differences and associated 95% confidence intervals (95% CI) were included in our analysis.

## Results

**Fig. 1:**
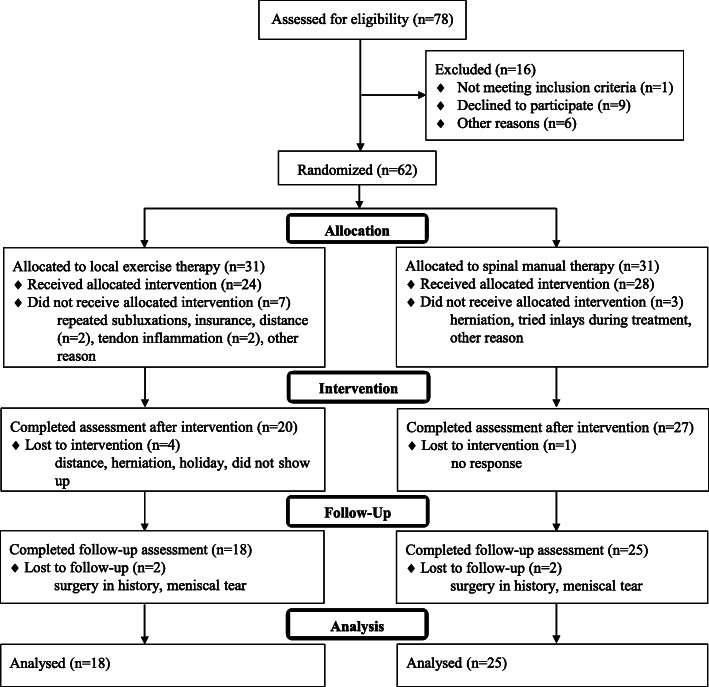
Flow diagram of the patients allocated to the local exercise and spinal manual therapy groups

Figure 1 shows the flow diagram of the progress through the phases of the parallel randomised trial of both intervention groups (that is, enrolment, allocation, intervention, follow-up, and data analysis). During the intervention some of the patients reacted aberrantly to treatment with increased knee pain and/or reduced function. For those patients additional medical imaging was requested by the therapists. The results of echography and/or MRI showed that 2 patients suffered from tendon inflammation (intervention) or developed a meniscal tear (follow-up). No participants reported harms directly related to the intervention (e.g. exercises or manipulations). All patients were asked for adverse events at every treatment session and were contacted by phone after the follow-up period.

The pre-intervention characteristics of the patients are shown in Table 1. There were no significant differences between groups, except for chondral lesions that were more common in the local exercise therapy group (*p* = 0.04).

**Table 1:** Comparison of baseline characteristics of study participants

Variable	Local exercise group (***n*** = 18)	Spinal manual therapy group (***n*** = 25)	***P*** value
Gender (% female)	15 (83%)	21 (84%)	0.95^a^
Age (years)	21 (17–34)	28 (21.5–31.5)	0.16^b^
Weight (kg)	71.8 ± 13.4	66.9 ± 8.6	0.15^c^
Height (cm)	172.8 ± 7.7	171.0 ± 7.7	0.57^c^
Duration of symptoms (months)	27 (19.5–80.3)	36 (24–90)	0.37^b^
Weekly participation in sport (% yes)	12 (67%)	17 (68%)	0.93^a^
Number of patients receiving physical therapy in the past (% yes)	14 (78%)	24 (96%)	0.07^a^
Number of patients with chondral lesions ≤ grade 2 (% yes)	8 (44%)	4 (16%)	0.04^a^
Number of patients with bilateral knee pain (% yes)	7 (39%)	8 (32%)	0.39^a^
Maximum pain* (mm)	73.6 ± 13.6	74.4 ± 14.3	0.85^c^
Minimum pain* (mm)	10.0 (4.3–22.5)	7.0 (0.0–17.0)	0.24^b^
Current pain* (mm)	27.0 (9.5–48.0)	33.0 (18.0–53.0)	0.68
AKPS	60.4 ± 10.6	66.9 ± 11.6	0.07
MVPF (N·m)	121.5 (105.0–154.5)	125.0 (103.3–137.8)	0.78

*n* number of patients, *%* percentage, mean ± standard deviation for normal distributed data, median (IQR) for non-normal distributed data, * pain measured using the visual analogue scale, *AKPS* anterior knee pain scale, *MVPF* maximum voluntary peak force, *N·m* Newton metre (torque), ^a^ chi-squared test, ^b^ Mann-Whitney U test, ^c^ independent t-test

### Primary outcomes

Significant differences for all pain outcome variables were observed between groups, in favour of the spinal manual therapy group (Table 2). After 6 weeks of intervention a minimal clinically important difference [37] of 23.4 mm in maximum pain was found [95% CI: 9.3, 37.6; effect size (ES): 1.04]. At 6 weeks of follow-up the between-group difference for maximal pain was 18.7 mm [95% CI: 1.4, 36.0; ES: 0.68]. In the local exercise therapy group maximum pain decreased significantly at 6 and 12 weeks compared to baseline, whereas all pain intensity variables improved significantly in the spinal manual therapy group.

**Table 2:** Post intervention comparison of pain, function and quadriceps peak force between the local exercise therapy and spinal manual therapy groups

	Local exercise group		Spinal manual therapy group		Between-group differences					
	6 weeks	12 weeks	6 weeks	12 weeks	6 weeks			12 weeks		
**Variables**	M	M	M	M	M	Effect size	*P* value ^c^	M	Effect size	*P* value ^c^
**Maximum pain (mm)**	59.7 ± 22.0 ^a^	52.7 ± 25.5 ^b^	36.3 ± 23.0 ^a^	34.0 ± 29.1 ^b^	23.4 [9.3; 37.6]	1.036	0.002	18.7[1.4; 36.0]	0.676	0.034
**Minimum pain (mm)**	6.5(0.0; 12.5)	1.5(0.0; 17.0)	0.0(0.0; 1.0] ^a^	0.0(0.0; 0.0) ^b^	6.5(0.0; 11.5)	−0.398	0.009	1.5(0.0; 17.0)	−0.304	0.046
**Current pain (mm)**	15.0(3.8; 28.5) ^a^	11.0(0.0; 31.8)	2.0(0.0; 11.5) ^a^	0.0(0.0; 14.5) ^b^	13.0(3.8; 17.0)	−0.307	0.044	11.0(0.0; 17.3)	−0.341	0.026
**AKPS**	70.9 ± 14.3 ^a^	75.6 ± 14.9 ^b^	83.3 ± 10.9 ^a^	87.1 ± 12.1 ^b^	− 12.4[− 20.2; − 4.7]	−1.000	0.002	− 11.5[− 19.9; − 3.3]	−0.869	0.008
**MVPF (N·m)**	140.0(111.0; 167.0)	133.5(110.3; 160.3)	127.0(117.0; 144.5)	128.0(112.0; 153.0) ^b^	13.0(− 6.0; 22.5)	−0.098	0.522	5.5(−1.7; 6.7)	−0.101	0.506

*M* mean [95% CI] ± standard deviation for normal distributed variables or median (IQR) for non-normal distributed variables, *AKPS* anterior knee pain scale (0–100), *MVPF* maximum voluntary peak force, ^a^ significantly different between baseline and 6 weeks (*P* < 0.025), ^b^ significantly different between baseline and 12 weeks (*P* < 0.025), ^c^ Bonferroni correction *P* < 0.017 accepted as statistically significant for between group comparison, effect size = Cohen’s d

Significant between-group differences were found for functionality, in favour of the spinal manual therapy group. Minimal clinically important differences on the AKPS both at 6 and 12 weeks after the start of the first intervention of − 12.4 [95% CI: − 20.2, − 4.7; ES: − 1.00] and − 11.5 [95% CI: − 19.9, − 3.3; ES: − 0.87] were found respectively. Compared to baseline the AKPS score improved significantly within both groups post-intervention.

### Secondary outcome

No significant between-group differences were found for MVPF. Compared to baseline peak force improved significantly only in the spinal manual therapy group after 6 weeks of follow-up (*p* = 0.021).

## Discussion

This is the first study supporting evidence that spinal manual therapy is more effective than local exercise therapy in patients with PFPS in the medium term. Compared to local exercise therapy, six sessions of manipulative therapy of the spine resulted in minimal clinically important differences in pain and functionality after 6 weeks of intervention and at 6 weeks of follow-up.

In the present study, pain intensity decreased significantly after intervention. These results are in line with previous findings supporting the use of exercise therapy in the treatment of patellofemoral pain in the medium term [38–40]. Since between-group differences were greater than the minimal clinically important difference of 20 mm for pain using a VAS after 6 weeks of intervention [37] it is suggested that spinal manual therapy is more effective than local exercise therapy. This finding supports the theory of the need of specific manual techniques aimed to achieve significant improvements in the model of regional interdependence in the treatment of patellofemoral pain [25, 41]. There is evidence that manipulations appear to modulate afferent input by stimulating inhibitory systems at various spinal levels [26]. As the knee (L2-S2), hip (L1-S4), lumbar zygapophyseal (L1-L5) and sacroiliac joints (L2-S3) share common nerve root levels, afferent information from a joint may induce a hypoalgesic effect in a peripheral structure related to its specific corresponding spinal level. Thus, altered central and peripheral pathways could be related to long lasting effects of interventions for PFPS. Because of the chronic character of PFPS, pain modulation mechanisms may deserve more attention in the treatment of patellofemoral pain [42, 43].

Function assessed by the AKPS improved significantly in both intervention groups at 6 and 12 weeks after baseline. These findings are supported by the work of Sahin et al. (2016) who reported functional gain in a sample of young females following combined knee and hip exercises. A between-group difference on the AKPS exceeding the minimal clinically important difference of 10 points was found post-intervention [37]. Since the spinal manual therapy group improved on average 6 points more than the local exercise therapy group, the between-group differences cannot be explained by the baseline differences (*p* = 0.07). The greater functional improvement in our manual therapy group was probably the result of greater pain reduction. Manipulation of the spine may have played a significant role in this improvement. Recent studies showed that manipulations of the lumbopelvic region contribute to earlier electromyography responses with higher amplitudes of gluteal muscles and vastus medialis muscles [10, 19, 24]. Likewise, long existing trigger points may impair adjacent joints [44]. Manipulations of these joints may deactivate these trigger points and may provide an improved control and coordination of muscles in patients with PFPS [45].

No differences between groups in MVPF of the quadriceps muscle at 6 and 12 weeks after the first intervention were found. However, compared to baseline only the spinal manual therapy group showed a small but significant improvement after 12 weeks. It has been suggested that individuals with PFPS experiencing pain can still deliver maximal quadriceps contraction [46]. However, delayed onset of vastus medialis oblique relative to vastus lateralis has also been shown in patients with PFPS [6, 47]. Therefore, it is assumed that quadriceps inhibition may be the resultant of acute pain rather than being related to the presence of a chronic condition like PFPS [48].

### Limitations

Firstly, a placebo effect responsible for the positive results on pain and function in the spinal manual therapy group cannot be ruled out. Patients were instructed to receive the best possible treatment for their long-lasting pain. All patients in the manual therapy group except one received exercise therapy in the past to treat their anterior knee pain. As such, these patients were not blinded to the type of intervention. Receiving an alternative treatment modality after receiving an unsuccessful standard treatment, may simply be sufficient to decrease pain intensity by increasing treatment expectations in some patients. Bialosky et al. (2011) [49] described that favourable outcomes of manual therapy may be related to the physiological and psychological effects of placebo. In combination with a possible positive patient-therapist interaction this may have influenced pain experience. Moreover, the local exercise therapy group showed smaller improvements in pain intensity compared to previous studies [50–52]. Since 81% of the patients already received local exercise therapy in the past, this might have resulted in a lack of motivation for this study. Future studies should therefore include a control group receiving sham manipulations to elucidate factors related to the placebo effect, like patient expectations of pain relief or treatment motivation [53]. Therefore, our results obtained in this relatively small convenience sample (*n* = 43) should not be extrapolated to the entire PFPS population.

Secondly, an experienced manual therapist assessed possible movement restrictions in the thoracolumbar spine, SIJ and hip joint. Joints with movement restrictions were manipulated afterwards. The assessment of hip joint restrictions has shown high inter-tester reliability [54, 55]. On the other hand, the inter-examiner reliability of passive assessment of segmental lumbar intervertebral motion is low [56]. Also, the diagnostic accuracy for SIJ dysfunction is poor because no widely accepted reference standard for SIJ dysfunction exists [57]. Therefore, the discriminative ability of our clinical examination to detect passive mobility restrictions of the spine may have been limited.

Finally, the follow-up period in our study was 6 weeks (medium term). As such, we did not assess the immediate nor the long-term effects of the interventions. The immediate positive effects of manipulations in patients with PFPS have been reported previously [11, 18, 58]. However, there is a need for future studies to examine the long-term effects of interventions by combining training programs with spinal manual therapy.

## Conclusion

Our results suggest that spinal manual therapy is more effective than local exercise therapy in improving pain and function in patients with PFPS in the medium term. Minimal clinically important differences of 23 mm for maximum pain and of 12 points for AKPS were found after 6 weeks of intervention, in favour of the spinal manual therapy group. These favourable effects persisted for 6 weeks after the last treatment. We suggest for future research to investigate whether combining local exercise therapy and spinal manual therapy is more effective than either single intervention on its own.

## Supplementary Information

**Additional file 1.**

## Data Availability

The datasets generated and/or analysed during the current study are available in the Onedrive repository of the corresponding author managed by the Vrije Universiteit Brussel. The datasets generated and/or analysed during the current study are not publicly available due compliance with GDPR requirements but are available from the corresponding author on reasonable request.

## Abbreviations

AKPS: Anterior knee pain scale
CI: Confidence interval
ES: Effect size
MVPF: Maximal voluntary peak force
PFPS: Patellofemoral pain syndrome
SIJ: Sacroiliac joint
VAS: Visual analogue scale
