# Supplementary material for: Effectiveness of local exercise therapy versus spinal manual therapy in patients with patellofemoral pain syndrome: medium term follow-up results of a randomized controlled trial
Source: BMC Musculoskelet Disord. 2021 May 15;22:446. doi: 10.1186/s12891-021-04310-9 (PMC8126114; doi:10.1186/s12891-021-04310-9)
Supplement: Supplementary file 1 — Additional file 1. [file 12891_2021_4310_MOESM1_ESM.docx]

**Effectiveness of local exercise therapy versus spinal manual therapy in patients with patellofemoral pain syndrome: medium term follow-up results of a randomized controlled trial**

Aldo Scafoglieri^1,2^, Jona Van den Broeck^1^, Stijn Willems^3^, Rob Tamminga^4^, Henk van der Hoeven^5^, Yde Engelsma^5^, Stijn Haverkamp^5^

1. Department of Physiotherapy, Human Physiology and Anatomy (KIMA), Experimental Anatomy Research Group, Vrije Universiteit Brussel, Laarbeeklaan 103, 1090 Brussel, Belgium
2. SOMT University of Physiotherapy, Softwareweg 5, 3821 BN, Amersfoort, The Netherlands
3. Department of Neuroscience, Vrije Universiteit Amsterdam, The Netherlands
4. Fysioholland, Medicort, Rijksweg 69, 1411 GE, Naarden, The Netherlands
5. Bergman Clinics BV, Rijksweg 69, 1411 GE, Naarden, The Netherlands

**Corresponding author**: Prof. dr. Aldo Scafoglieri, PT, MT, PhD, Department of

Physiotherapy, Human Physiology and Anatomy (KIMA), Experimental Anatomy Research Group, Vrije Universiteit Brussel, Laarbeeklaan 103, 1090 Brussel, Belgium, aldo.scafoglieri@vub.be, +3224774450

**Appendix 1: Description of interventions**

*Technique 1: Oscillation Techniques and connective tissue massage*


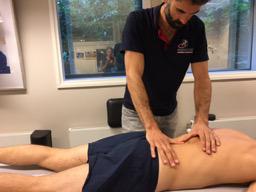
The oscillation techniques and connective tissue massage lasted maximum 10 minutes per treatment session. The oscillation techniques are performed with both hands / arms in the joint play of two vertebrae in the thoracolumbar region alternately in ventral, ipsi- and contralateral rotation. The patient is in prone position. The thenar and the hypothenar are placed on the arc of the underlying vertebra of the level to be treated on the ipsilateral side. Before performing the following manipulation(s), a connective tissue massage is performed to relax the tissues around the T12-L3 region.

*Technique 2 Traction-rotation manipulation of the Th12-L3 region*


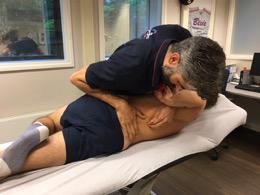
For the manipulation, the patient is in side position. Hip and knee of the lower leg are extended while the knee and hip in the upper leg are flexed in a 45 degrees angle. The spinal column is in ventral position with ipsilateral rotation. The hand of the underlying arm holds the wrist of the upper arm. The therapist is in front of the patient. The fixing hand / arm is placed under the patient's upper arm. The thumb is placed on the ipsilateral side against the spinous process of the upper vertebra of the level to be treated. The forearm rests against the ventral side of the patient's thorax. The wrist and middle fingers of the working hand / arm are placed on the contralateral side against the underlying vertebra's spinous process of the level to be treated. The forearm of the therapist rests against the dorsolateral side of the ilium. The fixing hand / arm maintains the position of the upper vertebra. The working hand / arm performs the traction / rotation manipulation. If no cavitation is observed, the manipulation is carried out in supine position (Appendix 1, technique 3)


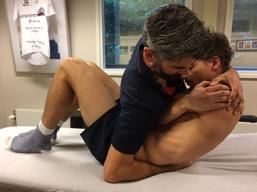
*Technique 3: Bilateral flexion gap manipulation of the Th12-L3 region*

The patient is supine position with bent knees. Both arms are crossed over the chest and are they hold the outside of the other shoulder. With the contralateral arm, the therapist holds the patient underneath the neck and the patient is raised so far that the therapist can fixate the underlying vertebrae of the Th12-L3 region with the ipsilateral arm while keeping the chest pressured on the elbows of the patient. Subsequently, a thrust is given from the upper body in dorsocranial direction to the upper vertebra to create a cavitation of the contralateral and ipsilateral side of the facet joint

*Technique 4: Manipulation of the SIJ*


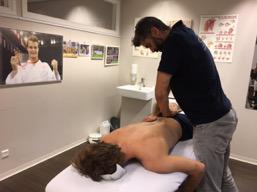
The patient is in prone position with the face to the side that is to be treated. The therapist is on the contralateral side with the homogeneous hand on the ilium and the heterogeneous hand on the contralateral and distal side of the sacrum. Through a body drop, the pulse is given in contranutation direction of the sacrum.

*Technique 5: Extension manipulation of the hip joint*


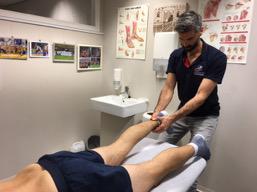
The patient is in prone position with the ankles over the treatment bench and with his head in a homonymous rotation. The therapist stands caudal from the patient with the fingers around the ankle. The hip is manipulated in the longitudinal direction of the leg.

*Technique 6: Mobilization exercise for the Th12-L3 region*


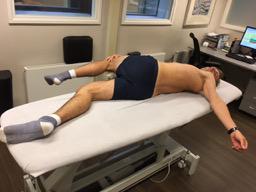
The patient is in supine position. The patient actively moves the left leg over the right leg so that the left shoulder rises. The head turns to the left side. The centre of rotation will thus be in the Th12-L3 region. This is also repeated for the other side. A total of 10 times is performed. This mobilization exercise was performed once a day at home by the spinal manual therapy group.


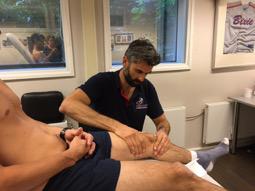
*Technique 7: Mobilization of the patella*

The patient is in supine position. The patella is mobilized in caudal and cranial direction at 0 degrees, 30 degrees and 60 degrees.

**Appendix 2: Maximum voluntary isometric contraction test for the quadriceps muscle**

After a 15-minute warming-up at an ergometer subjects were seated in the chair with the seat angle adjusted to give a maximum of 95° hip flexion. The position of the seat and other components of the dynamometer were standardized for each test. The trunk, lower back, and the leg were strapped to minimize compensation. The knee was resting in a 90 degrees angle. The lateral femoral condyle was used as a bony landmark for the axis of rotation. The subject is encouraged during the test.^1^ Each subject had a practice of 6 sub maximal repetitions at 90 degrees/second with the non-affected side followed by 1 min rest as part of the warm up prior to the test. After 3 maximal repetitions at 60 degrees/second for data collection the attachment of the dynamometer was changed to test the affected side.

**Appendix 3: Description of the physiotherapy intervention**

The physiotherapy intervention was based on evidenced-based hip and knee strengthening programs.^2,3^ Exercises were performed once a week in a session guided by a physical therapist. The following instructions were given:

- Do 3 sets of 10 repetitions with enough resistance that you are at 60-70% of your maximum strength. If it goes too easy, you may increase the elongation of your elastic resistance band or use a heavier elastic resistance band (color-coded Theraband was used to perform the resistance training: blue for men, green for women and red for girls).
- For the squat / leg press / single leg calf raises, you may use weights to increase the intensity of the exercise if it becomes too light. If you don’t have weights at home, you may use a backpack and fill it with weight, such as a bottle of water.

The intensity was adjusted every week under the supervision of the physiotherapist. Patients were also encouraged to increase the intensity of the exercises to gain muscle fatigue at the end of the set. Patients were finally trained to follow a home exercise program twice a week which consisted of the exercises in the Table hereunder , except for the leg press exercise.

| Exercise | Frequency |
| --- | --- |
| Knee extensions 90-45 degrees | 3x10 |
| Leg press 45 degrees | 3x10 |
| Squat 0-45 degrees | 3x10 |
| Single leg calf raises | 3x10 |
| Prone knee flexion with a resistance band | 3x10 |
| Hip abduction - sideways | 3x10 |
| Hip abductions with a restistance band - standing | 3x10 |
| Hip external rotations with a resistance band - seated | 3x10 |

**Appendix 4: Anatomical Map**

**
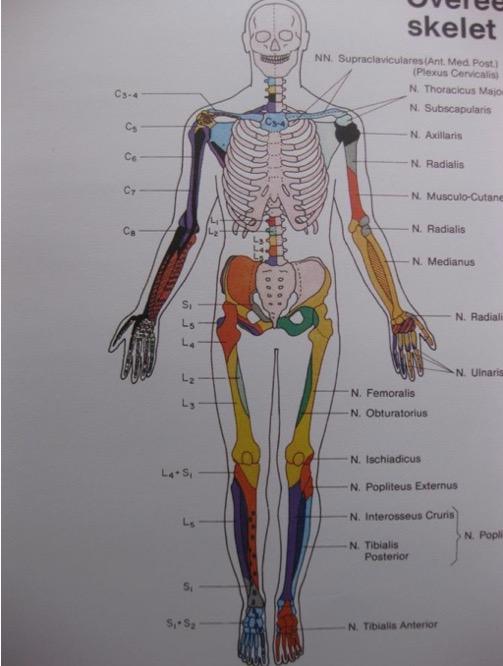
**

**Appendix 5: Exercise journal**

*Example of an exercise journal*

| *Week* | *Treatment received / exercise program at the clinic performed* | *Home exercises* performed day 1* | *Home exercises* performed day 2* |
| --- | --- | --- | --- |
| *1* |  |  |  |
| *2* |  |  |  |
| *3* |  |  |  |
| *4* |  |  |  |
| *5* |  |  |  |
| *6* |  |  |  |

** The home exercises are performed once a day for the manual therapy group (mobilisation exercise see Appendix 1, Technique 6) and each other day for the local exercise group (strengthening exercises see Appendix 3).*

**References**

1. Callaghan MJ, McCarthy CJ, Al-Omar A, Oldham JA. The reproducibility of multi-joint isokinetic and isometric assessments in a healthy and patient population. Clin Biomech. 2000;15:678-83.
2. Kooiker L, Van De Port IG, Weir A, Moen MH. Effects of physical therapist-guided quadriceps-strengthening exercises for the treatment of patellofemoral pain syndrome: a systematic review. J Orthop Sports Phys Ther. 2014;44:391-402.
3. Peters JS and Tyson NL. Proximal exercises are effective in treating patellofemoral pain syndrome: A systematic review. Int J Sports Phys Ther. 2013;8:689-700.
